# Leukocyte activation by (1→3)-β-D glucans

**DOI:** 10.1080/09629359791569

**Published:** 1997-11

**Authors:** Yoshiyuki Adachi, Mitsuhiro Okazaki, Naohito Ohno, Toshiro Yadomae

**Affiliations:** 1 Laboratory of Immunopharmacology of Microbial Products Tokyo College of Pharmacy 1432-1 Horinouchi Hachioji-shi Tokyo 192-03 Japan

## Abstract

We studied the activities of several kinds of β-glucans, including sonifilan, grifolan, *Sclerotinia sclerotiorum* glucan, laminarin and zymosan, on macrophages. Preculture of macrophages with inactive β-glucans rendered the cells unresponsive to subsequent stimulation with grifolan, suggesting a specific pathway in the β-glucan structure. The importance of protein C and phosphorylation of mitogen-activated protein kinase was demonstrated in the activation with grifolan or zymosan. Immunoprecipitation of complement receptor (CR3), coprecipitated other proteins carrying phosphotyrosine residues in stimulation with grifolan. These data suggest that protein kinase C and tyrosine kinases are essential for signal transduction, and that CR3 might participate in the activation through interaction with other intracellular proteins.


Mediators of Inflammation, 6, 251–256 (1997)

### Leukocyte activation by (1→3)-β-D-glucans

**Yoshiyuki Adachi[CA], Mitsuhiro Okazaki, Naohito Ohno and Toshiro Yadomae**

Laboratory of Immunopharmacology of Microbial Products, Tokyo College of Pharmacy, 1432-1 Horinouchi, Hachioji-shi, Tokyo 192-03, Japan

[CA]Corresponding author
Tel: +81 426 76 5599
Fax: +81 426 75 5570
Email: adachiyo@ps.toyaku.ac.jp

**We studied the activities of several kinds of β-glucans, including sonifilan, grifolan, *Sclerotinia sclerotiorum* glucan, laminarin and zymosan, on macrophages. Preculture of macrophages with inactive β-glucans rendered the cells unresponsive to subsequent stimulation with grifolan, suggesting a specific pathway in the β-glucan structure. The importance of protein C and phosphorylation of mitogen-activated protein kinase was demonstrated in the activation with grifolan or zymosan. Immunoprecipitation of complement receptor (CR3), co-precipitated other proteins carrying phosphotyrosine residues in stimulation with grifolan. These data suggest that protein kinase C and tyrosine kinases are essential for signal transduction, and that CR3 might participate in the activation through interaction with other intracellular proteins.**

**Keywords:** β-glucan, macrophage, tyrosine phophorylation

### Introduction

Macrophages are differentiated cells in the mononuclear phagocyte system [1]. They are widely distributed throughout the body and have a crucial function in defending the body against tumor cells or invasion by a wide variety of microorganisms. Fungal-derived (1→3)-β-D-glucans are known to activate a variety of macrophage functions [2–4]. In a previous study, we reported that grifolan, a gel-forming (1→3)-β-D-glucan from liquid-cultured mycelium of *Grifola frondosa*, induced antitumor activities against murine experimental tumors [5]. During the activation step, the importance of macrophages was suggested by a blockade of the macrophage phagocyte function when carrageenan was injected into the host [6]. Macrophages are also known as cytokine-producing cells, upon stimulation with products from various microorganisms [7]. It is clear that those cytokines activate the host defense function [8]. As glucans are able to activate the immune system and as cytokines play a key role in stimulating host defense activity, there is much interest in investigating the activity of β-glucans in cytokine production. We also studied signal pathways required for the macrophage activation induced by β-glucans. The signal pathway of the β-glucan-activated macrophage is discussed here.

### Materials and methods

### Mice

Male Institute for Cancer Research (ICR) mice were purchased from Japan S.L.C. (Hamamatsu, Japan) and used at 6–8 weeks of age.

### Reagents

Lipopolysaccharide obtained from *Escherichia coli* O111 and sodium orthovanadate were purchased from Sigma Chemical Co. (St Louis, Missouri, USA). H-7, HA1004, W-7 and W-5 were purchased from Seikagaku Kogyo Co. Ltd (Tokyo, Japan). Genistein and tyrphostin were purchased from Gibco-BRL Life Technologies (Grand Island, Maryland, USA). Herbimycin A (herbimycin) was purchased from Wako Pure Chemical Co. Ltd (Osaka, Japan).

### Preparation of β-glucans

Grifolan was prepared by the method described previously [5]. Sonifilan was donated by Kaken Pharmaceutical Co. Ltd (Tokyo, Japan). Laminarin and dextran were purchased from Sigma Chemical Co. and Seikagaku Kogyo Co. Ltd, respectively. Zymosan A and zymocel were purchased from Sigma Chemical Co. and Alpha-Beta Technology Inc. (Worcester, Massachusetts, USA), respectively.

### Culture of macrophage cell line

The mouse macrophage-like cell line RAW264.7 (Riken Cell Bank, Tsukuba, Japan) was cultured to confluence in Roswell Park Memorial Institute (RPMI) 1640/10% fetal calf serum. RAW264.7 cells were suspended at a cell density of $2.5 \times 10^6$ cells/ml in RPMI 1640/10% fetal calf serum. The cells were stimulated with or without grifolan (100, 500 mg/ml) or lipopolysaccharide (10 mg/ml) at 37°C for 24–48 h in a humidified 5% $CO_2$ incubator. After the incu-

© 1997 Rapid Science Publishers bation, culture supernatant was collected by centrifugation at 300 **g** for 5 min. Macrophages adhering to the culture plate were lysed in distilled water by repeated freezing and thawing (three times), followed by the addition of a twofold concentration of RPMI 1640 and filtration through a syringe filter unit (0.20 μm, Corning, New York, NY, USA).

### Preparation of murine peritoneal macrophages

Peritoneal macrophages were isolated from ICR mice, injected intraperitoneally with 2 ml proteospeptone 3 days before peritoneal lavage with 14 ml Hanks' balanced salt solution (HBSS; Nissui Pharmaceutical Co. Ltd, Tokyo, Japan) containing 5 mmol/l *N*-2-hydroxyethylpiperazine-*N*-2-ethanesulphonic acid (Sigma Chemical Co.), 100 U/ml penicillin, 100 mg/ml streptomycin (Meiji Seika Kaisha Ltd, Tokyo, Japan) and 5 U/ml heparin (Wako Pure Chemical Co. Ltd). The cells collected were washed twice with RPMI 1640 and then cultured in RPMI 1640 containing 1% heat-inactivated fetal calf serum. The cells were allowed to adhere to a 96-well culture plate (Sumitomo Bakelite Co. Ltd, Tokyo, Japan) for 3 h at 37°C in a 5% $CO_2$ incubator in 100 ml culture media. The culture was then washed twice with 100 ml/well of culture media to remove non-adherent cells.

### Determination of tumor necrosis factor (TNF)-α by enzyme-linked immunosorbent assay (ELISA)

For the ELISA system, a 96-well plate (MS 8596-F, Sumitomo Bakelite Co. Ltd) was coated with rat anti-mouse TNF-α monoclonal antibody (mAb; Pharmingen, San Diego, California, USA) in a bicarbonate buffer (pH 9.6). Uncoated binding sites in the wells were blocked with phosphate-buffered saline containing 0.25% bovine serum albumin (Biocell Laboratories, Carson, California, USA) and 0.05% Tween 20 (Wako Pure Chemical Co. Ltd) (BPBST). The wells were incubated with 50 ml of sample, culture supernatant or cell lysate in duplicate at 37°C for 40 min and then exposed to rabbit polyclonal antimouse TNF-α (Genzyme Co., Boston, Massachusetts, USA). The plate was developed using peroxidase-labeled goat antirabbit immunoglobulin (Ig)G (Organon Teknika, West Chester, Pennsylvania, USA) and peroxidase substrate (3,3′5,5′-tetramethylbenzidine microwell peroxidase substrate system; Kirkegaard and Perry Laboratories Inc., Gaithersburg, Maryland, USA). Aliquots of recombinant mouse TNF-α (R&D Systems, Minneapolis, Minnesota, USA) dissolved in 50 ml BPBST were used to construct a standard curve.

### RNA isolation and Northern blot analysis

Total RNA was extracted from $1.0 \times 10^7$ cells by the single-step guanidinium thiocyanate–phenol–chloroform method [9]. Total RNA (20 μg) was subjected to agarose–formaldehyde gel electrophoresis, blotted onto nylon filters, Hybond-N (Amersham Corp., Arlington Heights, Illinois, USA), and cross-linked by exposure to ultraviolet light for 5 min on each side. The filters were prehybridized in 50% formamide, 5 × saline sodium phosphate ethylenediaminetetraacetic acid (SSPE), 0.5% sodium dodecyl sulfate, 5 × Denhardt's solution, and 50 mg/ml salmon DNA for 4 h at 42°C. Overnight hybridization was performed at 42°C in the same buffer containing $2 \times 10^6$ cpm/ml of the TNF-α probes that had been radiolabeled with (α-$^{32}$P) deoxy (d)CTP (3000 Ci/mmol; Amersham) by the Megaprime DNA labeling system (Amersham). The TNF-α probe was a 1.3-kb EcoRI fragment in murine TNF-α plasmid DNA, pJT-1 (S. Natori, Tokyo University, Japan). This was followed by washes in 2 × SSPE, 0.1% sodium dodecyl sulfate at 45°C (2 × 15 min), in 1 × SSPE, 0.1% sodium dodecyl sulfate at 45°C (1 × 30 min) and then in 0.1 × SSPE, 0.1% sodium dodecyl sulfate at 45°C (1 × 30 min). The filters were exposed to the imaging plate at room temperature for 3 h. The autoradiographic signals were measured by a bio-imaging analyzer (BAS 2000; Fuji Film, Tokyo, Japan).

### Electrophoresis and Western blotting

Cellular extracts were subjected to sodium dodecyl sulfate–polyacrylamide gel electrophoresis as described previously [10]. After separation with 10% gels, the gels were washed and equilibrated in transfer buffer for 15 min and then electrotransferred to a polyvinyliden difluoride membrane. Immunoblotting of the membrane was performed after blocking non-specific binding by incubation with 1% bovine serum albumin and 0.05% Tween 20 in tris-hydroxymethyl-amino methane (TRIS)-buffered saline at pH 7.5. Detection was by a peroxidase-labeled antiphosphotyrosine recombinant antibody (RC20H; Transduction Laboratories, Lexington, Kentucky, USA) and a non-isotopic chemiluminescent system (ECL, Amersham).

### Immunoprecipitation of CR3 and its associating proteins

Macrophages were cultured with various stimuli, and then the cells were lysed with lysing buffer containing 20 mmol/l TRIS (pH 8.2), 140 mmol/l NaCl, 2 mmol/l ethylenediaminetetraacetic acid (EDTA), 1% Brij-58 (Pierce, Rockford, Illinois, USA), 5 mmol/l iodoacetamide, aprotinin, leupeptin (10 mg/ml), 1 mmol/l phenylmethylsulfonyl fluoride and 1 mmol/l sodium orthovanadate [11]. The lysate, which was preadsorbed with antimouse IgG antibody-conjugated agarose, was incubated overnight (rocking at 4°C) with anti-CR3 monoclonal antibody, and then antimurine IgG antibody-conjugated agarose. The precipitate was washed three times with lysing buffer and resuspended in 2 × Laemmli buffer (4% sodium dodecyl sulfate, 20% glycerol, 120 mmol/l TRIS, pH 6.8, and 0.01% bromophenol blue) with 12% 2-mercaptoethanol, boiled for 3 min and then electrophoresed through 10% sodium dodecyl sulfate–polyacrylamide gel. The proteins were transferred to the polyvinyliden difluoride membrane after electrophoresis, and the membrane was probed with RC20H as described above.

### Results

### Effect of various β-glucans on TNF-α production by macrophages *in vitro*

Since grifolan was able to stimulate macrophages to produce interleukin (IL)-1α, IL-6, and TNF-α *in vitro*, we investigated the question of whether other soluble and gel-forming glucans are able to induce similar cytokine production. We found that RAW264.7 cells cultured with 500 μg/ml of sonifilan, *Sclerotinia sclerotiorum*, laminarin and dextran for 24 h at 37°C did not induce any release of TNF-α (Table 1). This result was further confirmed by Northern blot analysis in which no expression of TNF-α messenger (m)RNA was observed in culture with sonifilan, laminarin and dextran (data not shown) [12]. These results indicated that grifolan is the only active gel-forming β-glucan tested in this study.

### Pretreatment effect of inactive β-glucans on grifolan-inducible macrophage activation

To establish whether or not the inactive glucans could be recognized by the macrophages, we investigated the effect of various β-glucans on grifolan-inducible TNF-α release by RAW264.7 cells. The cells were cultured with 250 μg/ml grifolan in the presence of 100, 250 or 500 μg/ml of sonifilan, *Sclerotinia sclerotiorum* glucan, laminarin and dextran at 37°C for 12 h, and the TNF-α concentration in each culture supernatant was quantified by ELISA. As shown in Table 2, TNF-α release was significantly reduced by the pretreatment with inactive glucans. Grifolan attaching or endocytosed by macrophages was also reduced by the pretreatment. It was therefore suggested that inactive β-glucans could be bound to the cell surface to which grifolan would interact to stimulate cytokine production.

### Effect of protein kinase C or calmodulin on TNF-α production induced with grifolan

To examine the intracellular signaling pathway for the TNF-α induction by grifolan, the expression level of TNF-α mRNA obtained from protein kinase C inhibitor- or calmodulin antagonist-treated cells was determined by Northern blotting analysis. H-7 or W-7 was used as the effective inhibitor for protein kinase C or calmodulin, respectively. The results are shown in Fig. 1.

With H-7, the mRNA of TNF-α was reduced to 40 μmol/l, while the negative control reagent, HA1004, did not affect the expression of mRNA. In contrast to the protein kinase C inhibitor, the calmodulin antagonist, W-7, did not reduce the mRNA level of TNF-α. These data suggest that protein kinase C has an important role in inducing TNF-α production.

**Table 1.** Effect of various glucans on tumor necrosis factor (TNF)-α production by macrophages *in vitro*. RAW264.7 cells were cultured with several concentrations of glucans for 24 h at 37°C in a humidified $CO_2$ incubator

| Glucans (500 μg/ml) | TNF-α (ng/ml) |
|---|---|
| Grifolan | 10.4 |
| Sonifilan | 1.4 |
| *Sclerotinia sclerotiorum* glucan | 0.5 |
| Laminarin | 1.1 |
| Dextran | 0 |

**Table 2.** Inhibitory effect (%) of β-glucans on tumor necrosis factor-α production induced with grifolan. RAW264.7 cells were cultured with a mixture of grifolan and the glucans indicated

| Glucans | 100 μg/ml | 250 μg/ml | 500 μg/ml |
|---|---|---|---|
| Sonifilan | 11.8 | 51.0 | 71.6 |
| SSG | 27.2 | 46.2 | 55.1 |
| Laminarin | 50.6 | 43.7 | 38.6 |
| Dextran | 0 | 0 | 4.4 |

SSG, *Sclerotinia sclerotiorum* glucan.

### Effect of inhibitors for tyrosine kinases on TNF-α induction by β-glucan

Tyrosine phosphorylation had also been reported as an important element in the signal transduction [13]. We therefore examined the pretreatment effect of tyrosine kinase inhibitors on TNF-α production. All inhibitors of tyrosine kinases showed a significant inhibitory effect on the production of TNF-α, implying that the activation pathways for this TNF-α production were regulated by tyrosine kinases (Fig. 2). To elucidate what phosphorylated protein was involved in the activation, we extracted the cellular proteins and subjected them to Western blotting analysis using antiphosphotyrosine antibody. Fig. 3 clearly demonstrates that mitogen-activated protein kinases have two different molecular weight enzymes, at 42 000 and 44 000, phosphorylated by stimulation with zymosan and grifolan. The results confirmed that tyrosine phosphorylation will be induced by cytokine-inducible β-glucans.

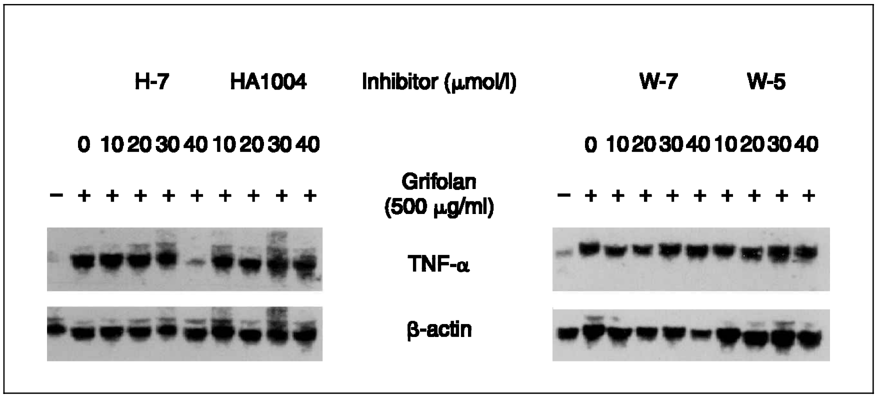

**Fig. 1.** Effect of signal transduction inhibitors on tumor necrosis factor (TNF)α messenger RNA. Expression was induced with grifolan. RAW264.7 cells were stimulated with grifolan (500 μg/ml) with and without inhibitors for 6 h at 37°C.

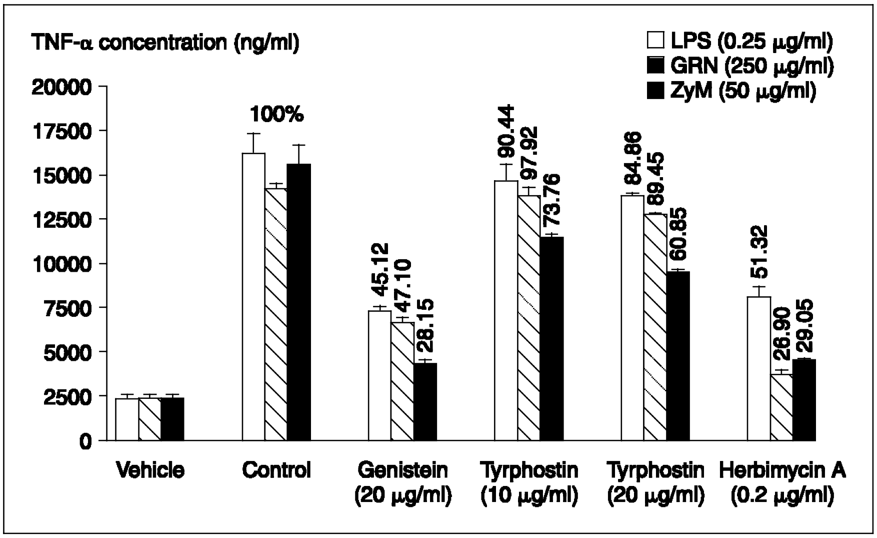

**Fig. 2.** Effect of tyrosine kinase inhibitors on the production of tumor necrosis factor (TNF)-α by RAW264.7. LPS, lipopolysaccharide; GRN, grifolan; ZyM, zymosan.

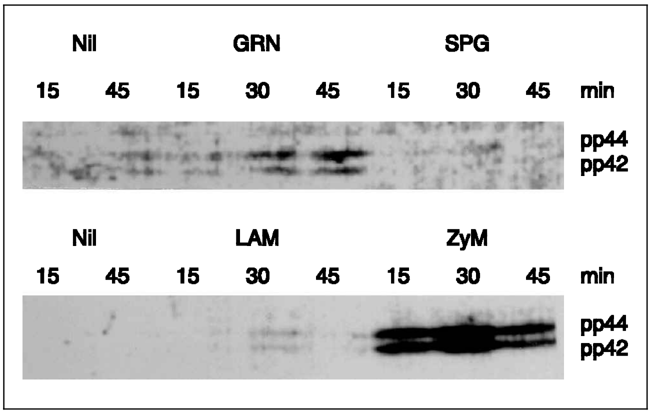

**Fig. 3.** Phosphorylation of mitogen-activated protein kinase in macrophage lysate induced with various stimuli. GRN, grifolan; SPG, sonifilan; LAM, laminarin; ZyM, zymosan.

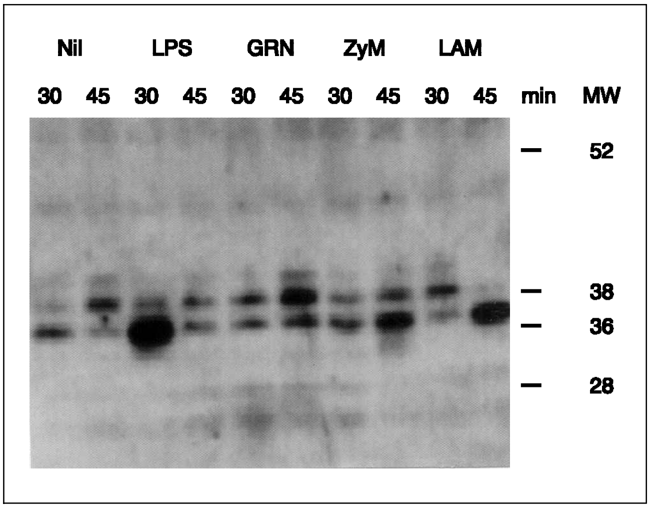

**Fig. 4.** Tyrosine phosphorylation of CR3-associating proteins. LPS, lipopolysaccharide; GRN, grifolan; ZyM, zymosan; LAM, laminarin.

### Tyrosine phosphorylation of 38 000 molecular weight protein associating with CR3

Recently, Ross and his colleagues [14] reported that complement receptor type 3 (CR3) was a β-glucan receptor. CR3 is a heterodimer transmembrane-type cell-surface molecule distributed on phagocytes such as macrophages and neutrophils [15]. The mechanism of the signal transduction generated by ligand-binding to CR3 is not fully understood. We tried to determine whether there are phosphorylated proteins associating with CR3 after the stimulation with β-glucans. The macrophages were incubated with several stimuli and then lysed with lysing buffer containing Nonidet P-40, various protease inhibitors and a phosphatase inhibitor. The cell lysate was immunoprecipitated with antimurine CR3 monoclonal antibody and antirat IgG agarose. The precipitation was loaded to sodium dodecyl sulfate–polyacrylamide gel electrophoresis and blotted on the polyvinyliden difluoride membrane. Phosphorylation was detected by POX-conjugated anti-phosphotyrosine monoclonal antibody. Biotinylated CR3 was precipitated with CR3 antibody, and the molecular weight showed 165 000 and 95 000 by incubation with POX-conjugated streptavidin (data not shown). When the membrane was probed with antiphosphotyrosine antibody, at least two positive bands emerged, at 38 000 and 36 000 (Fig. 4). In a comparison of the stimuli, lipopolysaccharide increased the phosphorylation of the 36 000 molecular weight protein with incubation for 30 min, whereas grifolan and zymosan induced phosphorylation of the 38 000 molecular weight protein. These results suggest that the β-glucans can increase the phosphorylation of the 38 000 molecular weight protein associating with CR3 molecules.

### Discussion

The present results show how β-glucans activate macrophages to produce various inflammatory cytokines with respect to the intracellular signaling pathway. Comparing structure–activity relationships in several β-glucans, we found that grifolan and zymosan were the only active β-glucans tested in this study. Zymosan has been reported to be a strong macrophage activator in both cytokine and superoxide production [16]. Although, the morphology of grifolan is not a particle form like that of zymosan, grifolan is a unique gel-forming active β-glucan on the cytokine induction.

When several inhibitors were used for tyrosine kinases and protein kinase C, the importance of those kinases for cytokine production was suggested. It seems that protein kinase C in particular is closely related to the expression of the TNF-α mRNA, while calmodulin is not. In the case of tyrosine kinases, grifolan induced phosphorylation of mitogen-activated protein kinase, which is known to be an important signal transduction element [13]. The recognition system for the β-glucans has recently been revealed as CR3 [14], one of the integrin molecules. Soluble, inactive β-glucans also bind to CR3, although those glucans had no effect on the cytokine production [14].

The present study showed that soluble β-glucans had an antagonistic effect on grifolan-induced TNF-α production. Grifolan might be effective for inducing activation of CR3 molecules by cross-linking the monomer CR3 to multimer. Petty and Todd [17] reported that activation of CR3 generated integration of a heteroreceptor molecule complex. It is possible that some adapter proteins in cytoplasm come together with the integrated CR3 and other receptor molecules. Our results show that activation of macrophages by grifolan induces such protein integration, and that those proteins are substrates for tyrosine kinases. Further work is needed to clarify the activation mechanisms in detail by stimulation with grifolan. However, this study provides some data for the understanding of immunopharmacological effects of β-glucans on the host defense.

### Conclusions

Grifolan has potent cytokine-inducing activity in macrophages. The intracellular pathway is affected by protein kinase C and tyrosine kinases, such as mitogen-activated protein kinase. Furthermore, CR3, a β-glucan receptor, might take part in the activation by associating with the substrate for tyrosine kinases.

### References

1. Auger MJ, Ross JA: The biology of macrophages. In: *The Macrophages*. Edited by Claire EL, James O'DM. New York: IRL Press; 1992:1–74.
2. Suzuki M, Tanaka H, Kinoshita A, Oikawa S, Osawa M, Yadomae T: Effect of orally administered β-glucan on macrophage function in mice. *Int J Immunopharmacol* 1990, **12**:675–684.
3. Sakurai, T Ohno N, Yadomae T: Intravenously administered (1→3)-β-D-glucan, SSG, obtained from *Sclerotinia sclerotiorum* IFO 9395 augments murine peritoneal macrophage function *in vivo*. *Chem Pharm Bull* 1992, **40**:2120–2124.
4. Yadomae T: Immunopharmacological activities of β-glucans: structure–activity relationships in relation to various conformations. *Jpn J Med Mycol* 1992, **33**:267–277.
5. Ohno N, Adachi Y, Suzuki I, *et al.*: Antitumor activity of β1→3-glucan obtained from liquid cultured mycelium of *Grifola frondosa*. *J Pharmco-Dyn* 1994, **9**:861–864.
6. Ohno N, Hayashi M, Suzuki I, *et al.*: Effect of activation or blockage of the phagocytic system on the antitumor activity of grifolan. *Chem Pharm Bull* 1986, **34**:4377–4381.
7. Nathan DF: Secretory products of macrophages. *J Clin Invest* 1987, **79**:319–326.
8. Revel M: Host defense against infections and inflammations: role of the multifunctional IL-6IFN-β2 cytokine. *Experientia* 1989, **45**:549–557.
9. Chomczynski P, Sacchi N: Single-step method of RNA isolation by acid guanidinium thiocyanate-PhOH-chloroform extraction. *Anal Biochem* 1987, **162**:156–159.
10. Laemmli VK: Cleavage of structural proteins during the assembly of the head of bacteriophage T4. *Nature* 1970, **227**:680–685.

11. Bohuslav J, Horejsi V, Hansmann C, *et al*.: Urokinase plasminogen activator receptor, β2-integrins, and Src-kinases within a single receptor complex of human monocytes. *J Exp Med* 1995, **181**:1381–1390.
12. Okazaki M, Adachi Y, Ohno N, Yadomae T: Structure–activity relationship of (1→3)-β-D-glucans in the induction of cytokine production from macrophages, *in vitro*. *Biol Pharm Bull* 1995, **18**:1320–1327.
13. Cano E, Mahadevan LC: Parallel signal processing among mammalian MAPKs. *Trends Biochem Sci* 1995, **20**:117–722.
14. Thornton BP, Vetvicka V, Pitman M, Goldman RC, Ross GD: Analysis of the sugar specificity and molecular location of the β-glucan-binding lectin site of complement receptor type 3 (CD11b/CD18). *J Immunol* 1996, **156**: 1235–1246.
15. Arnaout MA: Structure and function of the leukocyte adhesion molecules CD11b/CD18. *Blood* 1990, **75**:1037–1050.
16. Sanguedolce MV, Capa C, Bongrand P, Mege JL: Zymosan-stimulated tumor necrosis factor-α production by human monocytes: down-modulation by phorbol ester. *J Immunol* 1992, **148**:2229–2236.
17. Petty HR, Todd RF III: Receptor–receptor interactions of complement receptor type 3 in neutrophil membranes. *J Leukoc Biol* 1993, **54**:492–494.
